# Study on the Excretion of a New Antihypertensive Drug 221s (2,9) in Rats

**DOI:** 10.3390/ph18081138

**Published:** 2025-07-30

**Authors:** Yunmei Chen, Kuan Yang, Shaojing Liu, Lili Yu, Rong Wang, Bei Qin

**Affiliations:** 1 Xi’an Key Laboratory for Research and Development of Innovative Multi-Target Antihypertensive Drugs, Xi’an Innovative Antihypertensive Drugs International Science and Technology Cooperation Base, Xi’an Medical University, Xi’an 710021, China; yunmeichen@xiyi.edu.cn (Y.C.); yangkuan@xiyi.edu.cn (K.Y.); liushaojing@xiyi.edu.cn (S.L.); yulili@xiyi.edu.cn (L.Y.); wangrong@xiyi.edu.cn (R.W.); 2 Institute of Drug Research, Xi’an Medical University, Xi’an 710021, China; 3 College of Pharmacy, Xi’an Medical University, Xi’an 710021, China

**Keywords:** UPLC-MS/MS, excretion, hypertension

## Abstract

**Background/Objectives**: The novel compound 221s (2,9), derived from danshensu and ACEI-active proline, exhibits antihypertensive effects (50/35 mmHg SBP/DBP reduction in SHRs) with potential cough mitigation. However, its excretion kinetics remain unstudied. This study investigates 221s (2,9) elimination in rats to bridge this knowledge gap. **Methods**: Excretion of unchanged 221s (2,9) was quantified in urine, feces, and bile of Sprague-Dawley rats after oral administration (30 mg/kg). Concentrations of unchanged 221s (2,9) in all matrices were quantified using developed UPLC-MS/MS that underwent methodological validation. Excretion amount, excretion velocity, and accumulative excretion rate of 221s (2,9) were calculated. **Results**: Urinary excretion exhibited rapid elimination kinetics, reaching peak cumulative excretion rates (138.81 ± 15.56 ng/h) at 8 h post-dosing and plateauing by 48 h (cumulative excretion: 1479.81 ± 155.7 ng). Fecal excretion displayed an accelerated elimination phase between 4 and 8 h (excretion rate: 7994.29 ± 953.75 ng/h), followed by a sustained slow-release phase, culminating in a cumulative output of 36,726.31 ± 5507 ng at 48 h. Biliary excretion was minimal and ceased entirely by 24 h. Notably, total recovery of unchanged drug across all matrices remained below 1% (urine: 0.020 ± 0.021%; feces: 0.73 ± 0.069%; bile: 0.00044 ± 0.00002%) at 72 h. **Conclusions**: This study provides the first definitive excretion data for 221s (2,9). Quantitative analysis via a validated UPLC-MS/MS method revealed that fecal excretion is the principal elimination pathway for unchanged 221s (2,9) in rats, with direct excretion of the parent compound accounting for <1% of the administered dose over 72 h. Future studies will employ extended pharmacokinetic monitoring and concurrent UPLC-MS/MS analysis of the parent drug and phase II conjugates to resolve the observed mass imbalance and elucidate contributions to total elimination.

## 1. Introduction

Hypertension, commonly known as high blood pressure, is a prevalent chronic condition characterized by persistently elevated arterial blood pressure [1,2]. It is a significant public health issue affecting millions globally and is often referred to as “the silent killer” because it often develops without noticeable symptoms for years, even as it silently damages blood vessels, the heart, kidneys, and other organs [3].

The management of hypertension primarily focuses on pharmacological interventions, lifestyle modifications, and regular monitoring of blood pressure levels [4,5]. Angiotensin-converting enzyme inhibitors (ACEIs) are first-line antihypertensive drugs that exert their therapeutic effects by inhibiting the renin-angiotensin system [6]. However, ACEIs can cause adverse reactions such as irritative dry cough, angioneurotic edema, and renal dysfunction, significantly affecting the quality of life and health status of patients during treatment [7]. Therefore, there is an urgent need to develop ACEI derivatives with fewer side effects.

221s (2,9) is an innovative drug synthesized from danshensu, borneol, and proline, which is the active group of ACEIs, exhibiting significant antihypertensive activity [8,9,10]. Previous studies have demonstrated that 221s (2,9) exhibit no significant acute toxicity, with a maximum tolerated dose of 3000 mg/kg via oral gavage in mice. At this dosage level, no substantial effects were observed on body weight, dietary intake, or histopathological parameters of the liver, lungs, and kidneys [11]. 221s (2,9) reduces systolic blood pressure by 50 mmHg and diastolic blood pressure by 35 mmHg in spontaneously hypertensive rats (SHR). Notably, it significantly reduced serum levels of angiotensin II (Ang II) and angiotensin-converting enzyme (ACE), resulting in approximate reductions of 50 mmHg in systolic blood pressure and 35 mmHg in diastolic blood pressure in spontaneously hypertensive rats (SHR) [12]. The antihypertensive efficacy was comparable to that of captopril. Our preliminary data showed that 221s (2,9) reduces ACE content in murine serum, cerebral tissue, and renal compartments, suggesting its antihypertensive mechanism involves ACE inhibition. Notably, 221s (2,9) significantly decreases serum creatinine levels in spontaneously hypertensive rats (SHR) by 38.99% (*p* < 0.01) and downregulates renal NOX2 expression by 16.23% (*p* < 0.05). These findings indicate potential nephroprotective effects mediated through attenuation of oxidative stress pathways. Importantly, 221s (2,9) not only demonstrates favorable blood pressure-lowering effects but also exhibits potential organ protection, thereby maintaining therapeutic efficacy while mitigating the adverse effects commonly associated with ACE inhibitor drugs.

While the pharmacological efficacy of this novel antihypertensive agent in ameliorating hypertension has been demonstrated, knowledge regarding its elimination profile from the body remains uncharted territory. Understanding the excretion pathways of a novel compound like 221s (2,9) is critical for safety and efficacy [13]. It reveals elimination routes (e.g., renal, hepatic), determining dosing frequency, toxicity risks, and drug interaction potential [14,15]. This knowledge is essential for designing appropriate dosing regimens, especially for chronic antihypertensive therapy, and predicting necessary dose adjustments in patients with renal or hepatic impairment. To bridge the gap between the therapeutic potential of compound 221s (2,9) and its practical application, a comprehensive understanding of its pharmacokinetic behavior, particularly excretion kinetics, is imperative. Unfortunately, to date, no studies have ventured into investigating the excretion kinetics of 221s (2,9). Therefore, this study aims to fill this critical void by meticulously examining the excretion kinetics of 221s (2,9) in rats by using UPLC-MS. The findings from this investigation are anticipated to provide a scientific foundation for advancing 221s (2,9) towards clinical trials and, ultimately, offering a safer and more effective treatment option for hypertensive patients worldwide.

## 2. Results

### 2.1. Method Development

**Figure 1:**
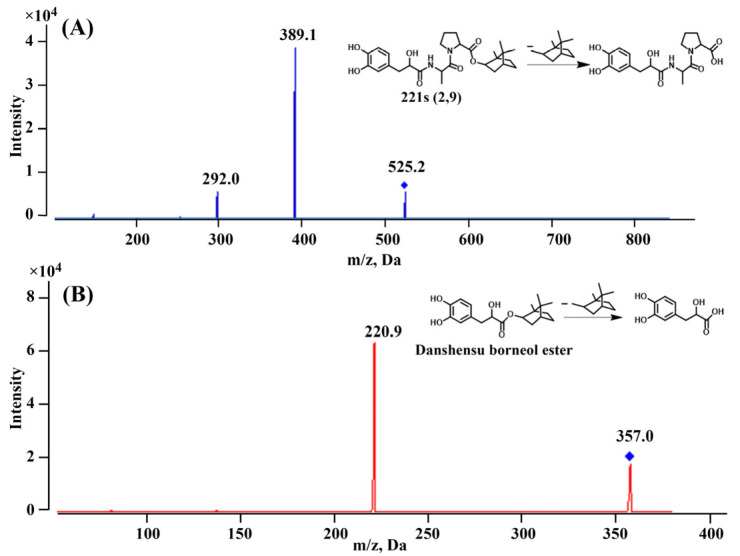
Product ion mass spectra of [M + Na]+ of (**A**) 221s (2,9) and (**B**) IS. The blue square refers to the parent ion in the mass spectrum.

As depicted in Figure 1, danshensu borneol ester was selected as the internal standard (IS) due to its chromatographic similarity to 221s (2,9). Preliminary full scans in both positive and negative ion modes revealed stronger 221s (2,9) signals in positive mode. Consequently, positive ion mode was employed, with 221s (2,9) and IS detected at *m*/*z* of 525.2 and 357.0, respectively. Following collision cell optimization, the most stable and least interfering product ions for 221s (2,9) (*m*/*z* of 389.1) and IS (*m*/*z* of 220.9) were identified. Finally, UPLC parameters (mobile phase, flow rate, column temperature, and column type) were optimized to achieve suitable retention times, high resolution, and enhanced sensitivity.

### 2.2. Method Validation

#### 2.2.1. Selectivity and Specific

**Figure 2:**
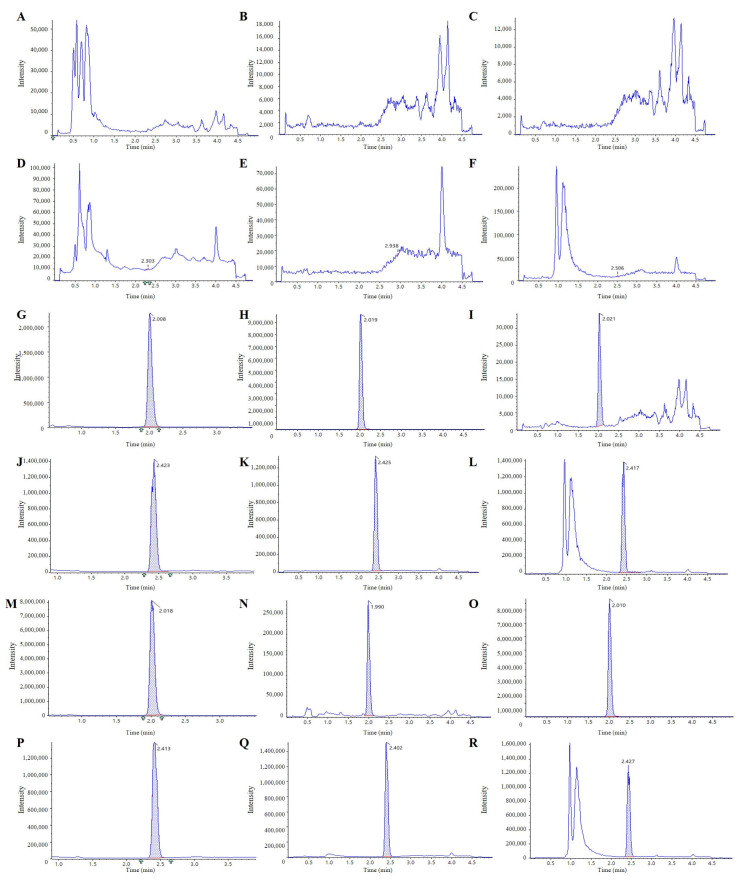
221s (2,9) in blank urine (**A**), feces (**B**), and bile (**C**), respectively; urine (**G**), feces (**H**), and bile (**I**) spiked with 2 ng/mL 221s (2,9), respectively; and urine (**M**), feces (**N**), and bile (**O**) samples collected 0–4 h after oral administration of 221s (2,9) (30 mg/kg), respectively. IS in blank urine (**D**), feces (**E**), and bile (**F**), respectively; urine (**J**), feces (**K**), and bile (**L**) spiked with 200 ng/mL IS, respectively; and urine (**P**), feces (**Q**), and bile (**R**) samples collected 0–4 h after oral administration of 221s (2,9) (30 mg/kg), respectively.

Blank matrices (urine, feces homogenate, and bile) spiked with or without analytes and IS, and dosed samples were processed and analyzed. Chromatograms (Figure 2) showed the IS and 221s (2,9) eluting at approximately 2.0 min and 2.4 min, respectively, in all blank matrices. Adequate resolution was achieved, and no interfering peaks were observed at these retention times.

#### 2.2.2. Linearity and LLOQ

The calibration curves for 221s (2,9) in urine, feces, and bile were established as follows: Urine: Y = 0.0073X + 0.1895 (r^2^ = 0.9995), range: 2–2000 ng/mL; Feces: Y = 0.0087X + 0.2421 (r^2^ = 0.9984), range: 2–2000 ng/mL; Bile: Y = 0.0095X + 0.1808 (r^2^ = 0.9972), range: 2–2000 ng/mL, where Y represents the peak area ratio of 221s (2,9) to the IS and X denotes the nominal concentration. The lower limit of detection (LLOD) was 2 ng/mL for all matrices, meeting the requirements for pharmacokinetic analysis.

#### 2.2.3. Precision and Accuracy

In the validation of 221s (2,9) analytical methodology, intra-day and inter-day precision/accuracy assessments were conducted across quality control samples spanning five concentration levels (2, 10, 500, 1600, and 2000 ng/mL). As summarized in Table 1, the urinary samples demonstrated acceptable performance with intra-day accuracy spanning −6.8% to 3.8% (RSD: 5.2–9.1%) and inter-day accuracy ranging from −6.4% to 4.1% (RSD: 2.6–9.5%). Fecal samples exhibited comparable reliability, showing intra-day accuracy deviations between −8.6% and 4.1% (RSD: 2.7–9.4%) alongside inter-day variations of −5.3% to 4.9% (RSD: 1.3–8.6%). The biliary matrix analysis revealed slightly broader fluctuations, with intra-day accuracy spanning −9.7% to 6.4% (RSD: 2.6–8.4%) and inter-day measurements ranging from −7.4% to 6.8% (RSD: 2.5–10.5%). Notably, all observed deviations remained within ±10% accuracy limits and 15% RSD thresholds recommended for bioanalytical method validation, demonstrating the method’s reliability for quantifying 221s (2,9) in these matrices.

#### 2.2.4. Extraction Recovery and Matrix Effect

As evidenced by the recovery rates and matrix effects data for 221s (2,9) presented in Table 2 and Table 3, all tested biological matrices (urine, feces, bile) demonstrated acceptable recovery efficiencies within validated ranges and exhibited negligible matrix interference effects, confirming the method’s suitability for quantitative determination of 221s (2,9) in complex biological matrices.

#### 2.2.5. Stability

Stability of 221s (2,9) was evaluated in urine, fecal, and bile matrices at low, medium, and high concentrations under diverse conditions: room temperature (2 h), three freeze–thaw cycles, −80 °C storage (30 days), 4 °C autosampler residence (24 h), and five-fold dilution. As detailed in Table 4, no significant degradation was observed in urinary/fecal/biliary samples. The obtained RSD ranges (urine: 3.3–9.4%; feces: 2.7–9.1%; bile: 1.6–7.6%) with all measured concentrations maintaining ±15% deviation from baseline values confirm the analytical method’s applicability for quantitative bioanalysis in complex biological specimens across various handling scenarios [16].

### 2.3. Excretion Study

**Figure 3:**
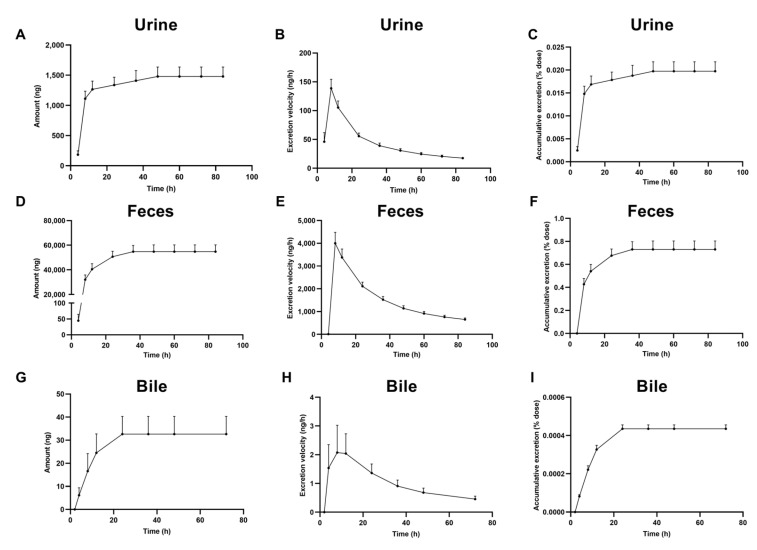
Excretion amount, excretion velocity, and accumulative excretion rate of 221s (2,9) in urine (**A**–**C**), feces (**D**–**F**), and bile (**G**–**I**) of rats following oral administration of 221s (2,9) at a single dose of 30 mg/kg (Mean ± SD, *n* = 6).

As shown in Figure 3, urinary excretion reached its peak cumulative excretion rate at 8 h post-dosing (138.81 ± 15.56 ng/h, Figure 3B), plateauing by 48 h (cumulative excretion: 1479.81 ± 155.7 ng, Figure 3A). This indicates rapid elimination predominantly during the early phase. Fecal excretion displayed an accelerated elimination phase between 4 and 8 h (excretion rate: 7994.29 ± 953.75 ng/h, Figure 3E), followed by a sustained slow-release phase (Figure 3D). Cumulative fecal output attained 36,726.31 ± 5507 ng at 48 h. Biliary excretion ceased after 24 h (Figure 3G), contributing minimally to the administered dose (0.00044 ± 0.00002%, Figure 3I).

The excretion profile of 221s (2,9) exhibited distinctive characteristics: fecal elimination was predominant (0.73% of administered dose), whereas biliary (0.00044%) and urinary (0.020%) excretion pathways contributed negligibly to total elimination. Although fecal dominance was observed, the overall mass balance remained below 1% of the administered dose. This discrepancy suggests either excretion of polar metabolites in conjugated forms that escaped detection by current analytical methods or the presence of delayed elimination processes not captured within the experimental timeframe. This discrepancy highlights the need for comprehensive metabolite profiling and extended pharmacokinetic monitoring to resolve the complete disposition pathway of 221s (2,9).

## 3. Discussion

The design of 221s (2,9) integrates two key modifications: Borneol, an upper ushering drug known for its ability to facilitate drug delivery across biological barriers, was strategically conjugated to the C-terminus of the proline residue via an ester linkage [17]. Danshensu was selected as the core component for its dual pharmacological benefits: demonstrated antihypertensive and organoprotective effects [18,19,20,21], coupled with its ability to chelate zinc ions within ACE’s catalytic center [22]. The strategic amide-bond conjugation of danshensu to the alanine-terminated proline residue potentially enables synergistic blood pressure reduction while conferring multi-organ protection across cardiovascular and renal systems.

The low excretion of 221s (2,9) can be attributed to two primary factors. First, the molecule contains ester bonds that are susceptible to hydrolysis in vivo. This enzymatic cleavage facilitates rapid metabolic transformation, reducing the proportion of unchanged drug available for direct elimination. Second, previous metabolite identification results (the data is being published) indicate that 221s (2,9) readily undergo phase II metabolic transformations in vivo, forming conjugated metabolites. The primary metabolites were mono-glucuronide, di-glucuronide, and sulfate conjugates. Integrating the current excretion data, we hypothesize that the compound is likely efficiently cleared via the biliary-fecal axis predominantly in the form of metabolites. This inference is based on the following rationale: phase II metabolites, due to their increased molecular weight, may be preferentially excreted via the biliary pathway [23,24]. Conjugated metabolites possess the characteristic of enterohepatic recirculation mediated by gut microbiota, which could explain the delayed phase observed in fecal excretion [25,26,27]. The current UPLC-MS/MS method was optimized solely for the parent drug, potentially leading to a systematic underestimation of metabolite excretion levels.

Future investigations will prioritize two complementary strategies to resolve the observed mass imbalance: prolonged pharmacokinetic monitoring through extended excretion sampling (up to 96 h post-dose) to detect potential secondary elimination peaks, coupled with concurrent development of UPLC-MS/MS methods enabling simultaneous quantification of both the parent compound and its major phase II conjugates. This integrated approach will clarify whether the sub-1% recovery stems from delayed excretion dynamics or analytical limitations in capturing polar metabolites, while establishing the relative contributions of each analyte to total elimination.

The minimal cumulative excretion of 221s (2,9) strongly suggests significant tissue accumulation, a phenomenon well documented in structurally analogous compounds. For instance, bornyl gallate—a borneol-conjugated derivative of gallic acid—exhibits pronounced renal accumulation alongside measurable brain distribution, attributed to the lipophilic borneol moiety enhancing blood-brain barrier permeability compared to the parent compound [28]. Such structural modifications often lead to altered pharmacokinetic profiles, including tissue-specific retention. Therefore, we hypothesize that 221s (2,9) may similarly undergo extensive tissue binding or intracellular uptake, potentially in organs with high metabolic activity or lipid content. Notably, plasma protein binding is frequently implicated in reduced drug excretion [29]. Consequently, comprehensive tissue distribution profiling and plasma protein binding assessment of 221s (2,9) constitute the primary focus of current investigative efforts.

While our current findings provide valuable insights into the excretion patterns of 221s (2,9), several limitations should be acknowledged. The potential influences of biological sex, dose-dependent effects, and multiple-dosing regimens on excretion kinetics remain uncharacterized. Consequently, future investigations will systematically examine sex-specific, age-dependent, and dose–response relationships governing the compound’s elimination pathways in both single- and multiple-dosing paradigms.

## 4. Materials and Methods

### 4.1. Chemical and Reagents

221s (2,9) (purity exceeding 99.5%) were chemically synthesized and supplied by the Xi’an key laboratory of multi synergistic antihypertensive innovative drug development (Xi’an, China). Danshensu borneol ester was generously contributed by the Key Laboratory of Resource Biology and Biotechnology in Western China from Northwest University (Xi’an, China). HPLC-grade solvents, including acetonitrile, methanol, and formic acid, were purchased from Thermo Fisher Scientific Co., (Beijing, China). HPLC-grade NH4F was purchased from Merck (Merck, Darmstadt, Germany). All other chemicals met HPLC quality standards.

### 4.2. UPLC-MS/MS Instrument and Conditions

According to previous data, analytical separation was performed using an ultra-performance liquid chromatography-tandem mass spectrometry (UPLC-MS/MS) platform comprising a Waters ACQUITY UPLC H-Class (Waters, Milford, CT, USA) system coupled to an AB Sciex Triple Quad 6500 mass spectrometer (AB Sciex, Boston, MA, USA). Chromatographic resolution was achieved on a Waters ACQUITY UPLC BEH C18 column (1.7 μm, 2.1 × 50 mm) maintained at 40 °C. The mobile phase system incorporated two components: solvent A (0.02% formic acid and 5 mM NH4F in deionized water) and solvent B (acetonitrile), delivered at 0.2 mL/min with a 10 μL injection volume. The gradient program was configured as follows: 55% solvent A (0.0–0.6 min), 5% solvent A (0.6–2.5 min), followed by re-equilibration at 55% solvent A (2.5–5.0 min).

Mass spectrometric detection utilized positive electrospray ionization (ESI+) in MRM mode, monitoring precursor-to-product ion transitions at *m*/*z* 525.1→389.1 (221s (2,9)) and 357.0→220.9 (IS). Instrument parameters were optimized as follows: curtain gas 25 psi, ion source temperature 500 °C, GS1/GS2 pressures 50/40 psi, ion spray voltage 5 kV, and collision gas pressure 10 psi. Compound-dependent parameter settings included decluttering potential (DP), entrance potential (EP), collision cell exit potential (CXP), and collision energy (CE) of 160/10/5/63 V for 221s (2,9) and 70/10/5/20 V for IS. Data processing was executed using Analyst software (v1.6.3).

### 4.3. Preparation of Calibration Standards and Quality Control Samples

Standard stock solutions of 221s (2,9) and IS were formulated in acetonitrile at identical concentrations (1 mg/mL). These stock solutions were then diluted with acetonitrile to create a range of working standard solutions. Quality control (QC) solutions were independently prepared from separate stock aliquots to ensure analytical independence. All solutions underwent cryopreservation at −20 °C for stability maintenance.

### 4.4. Method Validation

The optimized bioanalytical method for 221s (2,9) was fully validated in compliance with US FDA guidelines (2018, https://www.fda.gov/regulatory-information/search-fda-guidance-documents/bioanalytical-method-validation-guidance-industry (accessed on 27 May 2024)) in rat urine, feces, and bile. Assessments covered selectivity, specificity, linearity, LLOQ, precision, accuracy, extraction recovery, matrix effect, and stability.

#### 4.4.1. Extraction Recovery and Matrix Effect

Potential interference from endogenous components in rat urine, feces, and bile were investigated through selectivity/specificity assessments. Matrix samples from six rats (with/without 221s (2,9) and IS) were analyzed, and chromatographic peaks were evaluated according to retention times and MRM responses.

#### 4.4.2. Linearity and LLOQ

Linearity of 221s (2,9) in rat urine, feces, and bile was established using nine-point calibration curves. 221s (2,9)/IS peak area ratios were plotted against nominal concentrations with weighted (1/x^2^) linear regression, determining slope and R^2^. The LLOQ was defined as the lowest quantifiable curve concentration.

#### 4.4.3. Precision and Accuracy

Precision and accuracy were evaluated in rat urine, feces, and bile using five QC concentrations (2, 10, 500, 1600, and 2000 ng/mL). QC samples were analyzed in six replicates across three consecutive days (inter-day) and within the same day (intra-day). Accuracy (expressed as %RE = [(measured − nominal)/nominal] × 100) and precision (%RSD) required ≤15% deviation for all QCs except LLOQ (≤20%).

#### 4.4.4. Extraction Recovery and Matrix Effect

Extraction recovery was determined at three QC levels of 221s (2,9) (10, 1000, and 1600 ng/mL; *n* = 6) by comparing mean peak areas of pre-spiked QC samples versus post-extraction spiked blank matrices (urine/feces/bile).

Matrix effects were assessed via the matrix factor (MF), which was calculated as the ratio of peak area of 221s (2,9) in the extracted blank matrix (urine/feces/bile) to that in the working solution at three concentration levels (10, 1000, and 1600 ng/mL). For all matrices, MF values from six batches required ≤15% variability.

#### 4.4.5. Stability

The stability of 221s (2,9) in rat urine, feces, and bile matrices was evaluated using QC samples at three concentration levels (10, 1000, and 1600 ng/mL) across six replicates under four conditions per matrix: three freeze–thaw cycles, short-term storage (room temperature, 2 h), long-term storage (−80 °C, 30 days), and autosampler rack storage (4 °C, 24 h). Stability was quantified by comparing post-storage concentrations (normalized to freshly prepared QC samples analyzed in the same batch) with nominal values, with deviations ≤15% considered acceptable. Dilution reliability was assessed by 5-fold dilution of QC samples (1000/1600 ng/mL) in each matrix using blank rat plasma. Dilution accuracy was verified by comparing measured concentrations (post-dilution factor correction) to nominal values, with acceptable criteria defined as ≤15% relative error (RE) and ≤15% relative standard deviation (RSD).

### 4.5. Excretion Study

#### 4.5.1. Collection of Urine, Fecal, and Bile Samples

Six male Sprague-Dawley (SD) rats aged 8–10 weeks (body weight: 220–250 g) were individually housed in metabolic cages. Following a 12 h fasting period with free access to water, baseline urinary and fecal samples were collected as blank controls prior to drug administration. Samples of urine and feces were collected from 0–4 h, 4–8 h, 8–12 h, 12–24 h, 24–36 h, 36–48 h, 48–60 h, 60–72 h, and 72–84 h post-administration of 221s (2,9) with an oral dosage of 30 mg/kg. Urine samples were quantitatively measured for volume before storage at −80 °C while fecal samples were dried, pulverized, and weighed prior to preservation at −80 °C.

Six male SD rats were fasted for 12 h prior to drug administration, with free access to water. After being anesthetized with isoflurane, the rats were positioned supine on surgical platforms for common bile duct cannulation. Blank bile samples were collected following stabilization of bile flow. Following oral gavage of 30 mg/kg 221s (2,9), bile samples were sequentially collected during 0–2 h, 2–4 h, 4–8 h, 8–12 h, 12–24 h, 24–36 h, 36–48 h, and 48–72 h intervals. All biliary specimens were volumetrically measured and cryopreserved at −80 °C for subsequent analysis. Hydration maintenance was ensured through intraperitoneal administration of physiological saline during experimental procedures.

#### 4.5.2. Pretreatment of Urine, Fecal, and Bile Samples

A total of 10 μL of IS (1 μg/mL) was added to each urinary sample (100 μL), followed by the addition of three volumes of acetonitrile. The mixtures were vortexed and centrifuged twice. Then, the supernatants were concentrated, lyophilized and reconstituted in 100 μL of acetonitrile. The supernatants were subjected to instrumental analysis after final centrifugation.

Fecal samples (100 mg) were homogenized with three volumes of saline via ultrasonication for 10 min. Then, 10 μL of IS (1 μg/mL) was added, before adding three volumes of acetonitrile. The homogenate was spiked with 10 μL IS (1 μg/mL) and added with three volumes of acetonitrile. After vortexing and dual centrifugation, the supernatant was concentrated, lyophilized, and reconstituted in 100 μL of acetonitrile. The supernatants were subjected to instrument for analysis after final centrifugation.

Biliary samples (100 μL) were precisely aliquoted and added with 10 μL IS (1 μg/mL). Following homogenization, three volumes of acetonitrile were added to precipitate impurities. After vortexing and centrifuging twice, the supernatant was concentrated, lyophilized, and reconstituted in 100 μL of acetonitrile. The supernatants were subjected to instrumention for analysis after final centrifugation. Bile samples were processed identically to urine samples.

## 5. Conclusions

A validated UPLC–MS/MS method, characterized by simplicity, specificity, and sensitivity, was established for the determination of 221s (2,9) in rat urine, feces, and bile. Applied to an excretion study following oral administration, the method revealed fecal elimination as the principal pathway for the parent compound. Quantitative analysis revealed that direct excretion of unchanged drug accounts for <1% of the administered dose. Metabolite profiling studies corroborate the hypothesis that biotransformation products constitute the primary excretory forms, indicating extensive metabolic conversion prior to elimination.

## Figures and Tables

**Table 1:** Accuracy and precision of 221s (2,9) in rat urine, feces, and bile (*n* = 6).

	Normial Conc. (ng/mL)	Intra-Day		Inter-Day	
		Accuracy (RE, %)	Precision (RSD, %)	Accuracy (RE, %)	Precision (RSD, %)
Urine	2	−6.8	9.1	3.5	9.5
	10	2.4	6.3	−6.4	7.4
	500	2.9	8.4	−3.7	4.8
	1600	3.8	5.2	4.1	6.2
	2000	1.9	5.7	1.9	2.6
Feces	2	−8.6	9.4	−5.3	3.6
	10	−6.4	5.9	4.5	4.7
	500	3.4	5.7	4.4	3.2
	1600	4.1	2.7	4.9	8.6
	2000	−4.5	3.2	2.7	1.3
Bile	2	−9.7	7.5	−7.4	10.5
	10	−7.5	8.4	−4.9	8.4
	500	6.4	4.9	5.4	5.6
	1600	−3.8	5.1	6.8	7.2
	2000	3.1	2.6	3.3	2.5

**Table 2:** Extraction recovery of 221s (2,9) in rat urine, feces, and bile (*n* = 6).

	Spiked Conc. (ng/mL)	Matrix Effect (Mean ± SD, %)	RSD (%)
Urine	10	98.21 ± 7.51	7.6
	1000	101.65 ± 5.24	5.2
	1600	96.84 ± 8.35	8.6
Feces	10	99.14 ± 5.34	5.4
	1000	97.55 ± 4.28	4.4
	1600	103.27 ± 4.13	4.0
Bile	10	95.48 ± 6.22	6.5
	1000	102.47 ± 3.15	3.1
	1600	104.56 ± 1.68	1.6

**Table 3:** Matrix effect of 221s (2,9) in rat urine, feces, and bile (*n* = 6).

	Spiked Conc. (ng/mL)	Matrix Effect (Mean ± SD, %)	RSD (%)
Urine	10	89.15 ± 3.58	4.0
	1000	94.26 ± 5.49	5.8
	1600	100.52 ± 6.47	6.4
Feces	10	88.41 ± 10.44	11.8
	1000	99.75 ± 6.43	6.4
	1600	97.46 ± 5.28	5.4
Bile	10	88.39 ± 6.34	7.2
	1000	96.47 ± 3.44	3.6
	1600	99.25 ± 4.12	4.2

**Table 4:** Stability of 221s (2,9) in rat urine, feces, and bile (*n* = 6).

	Stability Conditions	Spiked Conc. (10 ng/mL)		Spiked Conc. (1000 ng/mL)		Spiked Conc. (1600 ng/mL)	
		RE (%)	RSD (%)	RE (%)	RSD (%)	RE (%)	RSD (%)
Urine	Room temperature for 2 h	−12.5	7.8	−1.5	3.3	3.4	4.0
	Three freeze/thaw cycles	−9.4	6.6	−6.4	8.7	−2.6	3.9
	−80 °C for 30 days	−8.7	5.3	−5.4	7.2	−5.5	6.7
	Autosampler rack (4 °C) for 24 h	−7.3	4.5	−6.3	6.8	3.9	3.4
	Dilution capability (factor: 5)	−12.8	3.4	−9.4	9.4	−7.4	8.1
Feces	Room temperature for 2 h	−6.7	4.8	−3.6	6.6	−4.7	5.5
	Three freeze/thaw cycles	−5.3	3.5	−4.8	4.9	−3.2	4.6
	−80 °C for 30 days	−6.4	4.1	−5.3	5.7	2.8	3.7
	Autosampler rack (4 °C) for 24 h	−4.9	5.8	−2.2	3.4	4.6	4.2
	Dilution capability (factor: 5)	−3.7	2.7	−3.7	4.1	−7.6	9.1
Bile	Room temperature for 2 h	−5.2	6.2	−4.5	6.5	5.1	5.8
	Three freeze/thaw cycles	−8.4	7.2	−4.1	5.7	4.2	6.2
	−80 °C for 30 days	−3.1	3.1	1.5	1.2	3.4	4.3
	Autosampler rack (4 °C) for 24 h	−4.4	3.8	−3.2	4.1	−3.8	3.7
	Dilution capability (factor: 5)	−5.6	7.6	2.4	3.3	−2.8	1.6

## Data Availability

The original contributions presented in the study are included in the article, further inquiries can be directed at the corresponding author.
